# The Origin of Protoconversation: An Examination of Caregiver Responses to Cry and Speech-Like Vocalizations

**DOI:** 10.3389/fpsyg.2018.01510

**Published:** 2018-08-24

**Authors:** Hyunjoo Yoo, Dale A. Bowman, D. Kimbrough Oller

**Affiliations:** ^1^Department of Communicative Disorders, The University of Alabama, Tuscaloosa, AL, United States; ^2^Department of Mathematical Sciences, University of Memphis, Memphis, TN, United States; ^3^Institute for Intelligent Systems, University of Memphis, Memphis, TN, United States; ^4^School of Communication Sciences & Disorders, University of Memphis, Memphis, TN, United States; ^5^Konrad Lorenz Institute for Evolution and Cognition Research, Klosterneuburg, Austria

**Keywords:** turn-taking, mother-infant interaction, speech-like vocalizations, protophones, cry, LENA, distress vocalizations, newborns

## Abstract

Turn-taking is a universal and fundamental feature of human vocal communication. Through protoconversation, caregivers play a key role for infants in helping them learn the turn-taking system. Infants produce both speech-like vocalizations (i.e., protophones) and cries from birth. Prior research has shown that caregivers take turns with infant protophones. However, no prior research has investigated the timing of caregiver responses to cries. The present work is the first to systematically investigate different temporal patterns of caregiver responses to protophones *and* to cries. Results showed that, even in infants' first 3 months of life, caregivers were more likely to take turns with protophones and to overlap with cries. The study provides evidence that caregivers are intuitively aware that protophones and cries are functionally different: protophones are treated as precursors to speech, whereas cries are treated as expressions of distress.

## Introduction

### Overview of the present effort

The importance of early caregiver-infant interaction in cognitive, social and language development has been well-documented for decades (Ainsworth and Bell, 1974; Beckwith et al., 1976; Bakeman and Brown, 1980; Tomasello, 1992; Murray et al., 1996; Jaffe et al., 2001; Feldman, 2007a,b; Bornstein and Bruner, 2014). The research has emphasized the sense in which early turn taking vocal interactions provide a basis for emotional bonding (Bell and Ainsworth, 1972; Blehar et al., 1977; Ainsworth, 1979; Keller et al., 1996; Völker et al., 1999), a protoconversational frame, and a foundation for sociality and for speech communication (Bateson, 1975; Trevarthen, 1977; Tronick et al., 1980; Papoušek, 1995; Goldstein et al., 2003). However, there has been a remarkable gap in this literature in that it has ignored the timing of caregiver responses to infant cries, focusing instead on timing of contingent patterns of response to speech-like vocalizations (i.e., protophones, Oller, 2000). The gap is especially notable considering the fact that infants produce *both* protophones and cries from birth (Keller and Schölmerich, 1987; Nathani et al., 2006; Dominguez et al., 2016; Jhang and Oller, 2017). Stern et al. (1975) speculated that caregiver responses to cry would tend to overlap rather than alternate. Empirical research on this previously unstudied speculation is important because it could illustrate that caregivers express an intuitive awareness of protophones as potential speech material by taking turns with them, while at the same time treating cries differently, speaking over them. The present study aims to systematically investigate timing of caregiver utterances in response to both protophones and cries.

Both very early precanonical protophones and later canonical syllables are foundations for speech (Oller, 1980, 2000; Koopmans-van Beinum and van der Stelt, 1986; Oller et al., 2013). However, compared to canonical syllables, precanonical protophones show far less obvious speech-like characteristics. The present research targets caregiver responses to the earliest precanonical protophones at 0, 1, and 3 months of age, affording the opportunity to evaluate the possibility that caregivers intuitively know protophones are precursors to speech even from the first months of life and treat them as such in the earliest interactions.

The research provides a new perspective on caregiver-infant interaction, because the data are derived from all-day recordings in infant homes. Prior research has almost entirely been conducted in structured settings where caregivers and infants have been expected to interact for the recordings. In these settings, with caregivers and infants always in the same room, caregivers have usually responded to infant vocalizations at very high, and presumably unrepresentative rates (see review in Fagan and Doveikis, 2017). Our approach should provide a more representative portrayal of both rates and timing of interactions.

### Vocal turn-taking in conversation

In conversation, human adults contingently interact with each other and overwhelmingly take turns (Sacks et al., 1974; Sidnell and Stivers, 2012; Clayman, 2013; Hayashi, 2013; Abney, 2016). Levinson (2016) has suggested several reasons why investigating the turn-taking system in conversation is important both in adults and in parent-infant interaction, and thus why the turn-taking system has drawn increasing attention in the field of psycholinguistics and conversation analysis. The turn-taking system has universal characteristics that allow researchers to evaluate human predispositions and capabilities that are fundamental to language acquisition and language processing (Levinson and Torreira, 2015; Levinson, 2016). However, it has been frequently reported, particularly in the field of anthropology, that there are culture-specific features in human communication (Stross, 1972; Brown, 1998; Tanaka, 1999). For example, although systematic quantification has not been provided, speakers in the Nordic countries have been reported to be relatively silent and to tend to interpose long silences between turn transitions. Long silences between turns may require “tolerance of silence” in American speakers (Lehtonen and Sajavaara, 1985, p. 279). Gender-specific features have also been investigated (Maltz and Borker, 1982; Coates, 1994, 1997). The cited research indicated that female friends were more likely to overlap or take turns without a gap than male friends. In other words, the collaborative floor (termed the “all-in-together mode”) was found to be more common in conversations between female friends.

Not only adult communication, but also caregiver-infant communication has been investigated to examine cross-cultural variations. Indeed research has suggested that features of parenting or caregiver-infant interaction vary cross culturally (Fogel et al., 1988; Richman et al., 1992; Rabain-Jamin and Sabeau-Jouannet, 1997; Keller et al., 2005; Kärtner et al., 2010). For example, Rabain-Jamin and Sabeau-Jouannet (1997) reported that French mothers tended to interact with their infants in dyads whereas Senegalese mothers (Wolof speaking) frequently included additional conversational partners.

However, a growing body of research has reported relatively universal characteristics of human interaction, particularly focusing on rapid turn-taking (Stivers et al., 2009; Heldner and Edlund, 2010). For example, Stivers et al. (2009) have provided empirical evidence reporting that speakers in 10 different languages (including the Nordic countries) showed similar latencies (around 250 ms) in response to questions, although there were subtle differences across languages. Wilson and Wilson (2005) also claimed that turn-taking patterns are similar regardless of cultures or social classes.

Rapid turn-taking between conversational partners is a remarkable feature given that one must comprehend, plan to produce and predict when to begin talking, while listening to the other's speech (Levinson, 2016). Obviously, rapid turn taking between speakers requires quick cognitive processing, considering that it takes at least 600 ms to prepare a single word production (Indefrey and Levelt, 2004; Indefrey, 2011). Sacks et al. (1974) systematically characterized turn-taking as a primary pattern in conversation. Other researchers have reported timing (or lags) of turn-taking, indicating that short latencies within hundreds of milliseconds are overwhelmingly common in conversation (Heldner and Edlund, 2010; Levinson and Torreira, 2015). Recent studies have attempted to examine the complex cognitive processing (e.g., prediction of the end of the utterance) that occurs in preparation for rapid turn transitions. Bögels and Levinson (2017) reviewed neurocognitive studies (e.g., brain imaging and electroencephalography) showing that listeners immediately recognized speech acts (such as statements or questions) and planned to produce speech for the next turn while listening.

To demonstrate that the turn-taking system is fundamental to human communication, it is important to investigate whether caregivers and infants show similar turn-taking patterns in vocal interaction (Levinson, 2016). If turn-taking occurs in the earliest interactions, does it show timing similar to that of more mature interactions? Addressing this question will help clarify how conversation emerges in development. And by considering possible differences in timing of parent responses to cries and protophones, we may illuminate the nature of parent awareness of the protophones as potential conversational material very early in life.

It is noteworthy, of course, that turn-taking is not the only way that speakers interact. Sometimes speaking in unison occurs both in adult conversation and in parent-infant interaction (Stern et al., 1975). The function of speaking in unison has been speculated to be associated with various circumstances, including high arousal expressions of coordinated action/thinking or of discord. In the present work, the analysis focuses only on the extent to which unison (or overlapping vocalization) and alternation between parents and infants reflects differences in how parents react to cries and protophones in the first 3 months of infant life. Ultimately of course it will be desirable to address the functions of overlapping and alternating talk as well as nonverbal behaviors under a single umbrella of theory that differentiates a wide variety of possible functions of coordinated rhythms in interaction.

### Development of the turn-taking system: focus on the protophones

Early caregiver-infant vocal interaction has been reported to surprisingly resemble conversation in mature languages (Bateson, 1975; Jasnow and Feldstein, 1986; Papoušek, 1995). Caregiver-infant interaction has been investigated for decades because it has been suggested to influence infant cognitive, emotional, and language development (Bloom et al., 1987; Jaffe et al., 2001; Goldstein et al., 2003, 2009). Researchers have provided evidence that even before speech, caregivers and infants show turn-taking patterns, and this vocal interaction in early infancy has been called “protoconversation” (Bateson, 1975; Trevarthen and Aitken, 2001). For example, Bateson (1975) showed early mother interaction with infants as young as the second month of life in various modalities including gaze and vocalization. After Stern et al. (1975) suggested two different modes of communication in mother-infant dyads, representing coaction (simultaneous or overlapping talk) and alternation (turn taking), researchers attempted to find a transition between the two. It was seemingly assumed by some that there might be a developmental trajectory of the two modes in dyads, with coaction preceding alternation. Similarly it seemed to be assumed that the mother might be primarily responsible for the appearance of vocal interaction at the youngest infant ages, while the infant might need to learn to be an active turn-taker (Miura et al., 2007; Ishihara et al., 2009). To explain how the mother could create the appearance of bilateral interaction at very young ages, consider the possibility that she can anticipate the *offset* of infant utterances (that are produced endogenously) and respond to them, and further that she can anticipate the *onset* of infant utterances and speak before them. In one study, vocal turn-taking was reported to be increased between 12 and 18 weeks of age after overlapping between 7 and 13 weeks (Ginsburg and Kilbourne, 1988). This study has been cited many times in an attempt to argue that infants are more likely to overlap with caregivers in early months and gradually to develop turn-taking capability. The study has sometimes been interpreted to suggest that the mother drives (with limited success) most of the apparent interaction at the youngest ages, and that the baby learns to interact actively with experience, resulting in more consistent alternation of mother and infant voices at older ages. Interpretation of the study is, however, hampered by its small number of dyads (3) and high variability among them, as well as the small number of interactive samples and range of circumstances of interaction that were observed.

A recent study attempted again to investigate developmental trajectories of turn-taking in caregiver-infant interaction. Hilbrink et al. (2015) investigated developmental trajectories of mother-infant interaction with infants ranging from 3 to 18 months of age. The authors reported that infants between 3 and 5 months produced more than 40% of their turns in overlap with caregivers, while this proportion of overlap decreased after 5 months and dropped to around 20% at 18 months. Turn-taking patterns were present from 3 months through 18 months, and only gap durations were different depending on ages. Gratier et al. (2015) also attempted to investigate developmental courses and showed that around 30% of infant vocalizations involved in turn-taking were overlapped with maternal vocalizations both at 8–13 weeks and at 17–21 weeks. In the Gratier et al. work, turn-taking patterns did *not* increase in older infants. Lavelli and Fogel (2002) conducted a longitudinal study on communication through gaze and facial expression between 1 and 14 weeks and found significant developmental changes around 2 months. The authors emphasized that critical neurodevelopmental changes occur at 2 months of age, and that most studies on turn-taking have investigated infants after this critical period. We note the important exception of Dominguez et al. (2016) who recently focused on infants at 2 to 4 days of age. These authors reported that 32% of infant vocalizations were overlapped with mothers' vocalizations. Surprisingly, when infants produced vocalizations that followed maternal vocalizations, about 70% were produced within 1 sec, the same time frame typical of older ages.

Taken together, researchers have reported consistent results in terms of presence (or early emergence) of turn-taking in protoconversation, even though many infant vocalizations are overlapped with maternal vocalizations (Bateson, 1975; Elias et al., 1986; Beebe et al., 1988; Gratier, 2003; Hsu and Fogel, 2003). However, the evidence is not conclusive about whether turn-taking increases and overlap decreases as a function of age. In addition, Stern et al. (1975) suggested that both coaction and alternation exist throughout life for different communicative functions, and thus coaction does not necessarily reflect an immature pattern of interaction. Their suggestion creates possibilities that interaction patterns may be different depending on functions of vocalizations. However, surprisingly, almost all prior research on early turn-taking has focused only on protophones and has ignored responses to cries.

### Limitations in prior research: the failure to compare responses to protophones and cries

Since language is primarily vocal, a key question in how vocal interaction develops concerns the nature of infant vocalizations themselves. We emphasize the distinction between early cries and vocalizations deemed to be precursors to speech, the protophones. One might imagine that these sounds would have been systematically differentiated in the study of early vocal interaction. In fact as far back as Stern et al. (1975), it has been speculated, but not quantified, that caregivers may tend to speak simultaneously with cry as opposed to non-cry. Yet, despite decades of research in early caregiver-infant interaction, as far as we know, no prior research has explicitly provided a clear definition of distress vocalizations (e.g., fusses and cries) as opposed to protophones, and consequently no research has differentiated caregiver responses to these importantly different kinds of sounds. Instead, it has been simply mentioned in some research that infant distress/negative sounds (e.g., fusses, whimpers, and cries) were excluded (e.g., Hsu and Fogel, 2003; Gratier et al., 2015). In other cases distress and non-distress sounds appear to have been grouped together without clear information about what the definitions were and how groupings were established (e.g., Bell and Ainsworth, 1972). Therefore, it has not been possible to determine what sounds have been included in most caregiver-infant interaction analyses.

In addition, although infants produce both cries and protophones from birth (Nathani et al., 2006), most research appears so far to have attempted to investigate caregiver-infant interaction exclusively with speech-like sounds, which they have generally termed “non-distress” sounds (e.g., Hsu et al., 2001). Kaye and Fogel (1980) treated distress sounds somewhat differently from other studies, mentioning that “less extreme fussiness was considered a normal part of the interaction” (p. 455). Still, the authors' criteria for identifying fussiness were vague. In the absence of clear definitions (differentiating non-distress vocalizations as opposed to distress vocalizations), it is not clear exactly what sounds have been included under the heading “non-distress.”

We propose that a clear distinction between protophones and distress sounds is critical for the study of caregiver-infant vocal interaction because it makes sense (in accord with the opinion of Stern) to imagine that caregivers will interact differently with the different sounds, since protophones are presumable precursors to speech (and are thus amenable to conversation), while distress sounds may be antithetical to conversation. It is nonetheless important to recognize that infant cries can play a role in establishing attachment with caregivers, which is fundamental to infant social, cognitive, and language development (Bell and Ainsworth, 1972; Ainsworth and Bell, 1974; Sroufe and Waters, 1977). Thus, it makes sense to explore caregiver-infant interaction with *both* protophones and cries.

Another key limitation in prior studies on caregiver-infant interaction is that they have been overwhelmingly conducted in artificial structured settings (either in a laboratory or home). Mothers have been asked to interact with her infants with (or without) staff observing only during a brief artificially designed period, usually less than 10 min (review in Fagan and Doveikis, 2017). In such structured settings (with staff observing during brief periods), mothers and infants may not interact naturally, and thus it may not be possible for researchers to obtain representative data. While interaction in well-defined laboratory circumstances is a legitimate target for research, it is also important to evaluate vocal interaction in the totally natural environment of the home. In that environment there are many differences from laboratory sampling. For example, parents are often not in the same room with infants at home, whereas in laboratory research they are usually in the same room with the infant and are expected to interact face-to-face. There is presumably a much reduced such expectation in the context of all-day home recordings. The purpose here is not to *compare* parent-infant interaction between structured and naturalistic settings but merely to present data from all-day home recordings, which we presume to provide a maximally naturalistic characterization that may reflect more representative and valid interactions.

### Rationale for the present study

In the present study, we pursued the question of the origin of vocal interactivity by investigating the timing of caregiver vocalizations in the hope of illuminating whether (or how) caregivers play a role in controlling or scaffolding vocal interaction. Infants produce both protophones and cries from birth and those vocalizations operate as vehicles for possible interaction with caregivers. Protophones are known to be precursors to speech while cries express distress. Our study evaluates, for the first time, the relative timing of caregiver vocal responses to protophones and distress sounds (e.g., cries and whimpers)^1^. If caregivers tend to take turns with protophones, while speaking simultaneously with cries and whimpers, we can argue that caregivers intuitively treat protophones in a way that allows infants to begin to learn about conversation. Research has so far failed to show caregivers' systematic responses to protophones as opposed to cries because prior research has largely ignored caregivers' interaction with cries. Moreover, no prior interaction research has provided systematic and clear criteria for identifying distress as opposed to protophone sounds.

We investigated timing of caregiver vocalizations in response to infant protophones as opposed to cries specifying acoustic/auditory criteria to differentiate protophones from cries. In addition, to evaluate the origins of the human tendency and learning pattern for interactivity, we sought representative data from the natural interactive setting. We made all-day recordings in the home and selected periods with naturally-occurring high volubility and interactivity. Our approach allowed sampling from entire days of home recording. By using this approach, we hoped to provide maximally representative data on vocal interaction, and to illuminate the beginnings of human conversation.

## Methods

### Participants

12 infants contributed data for the present study: 9 infants at 0 months and 10 infants at both 1 and 3 months. Among the 12 infants, 7 were fully longitudinally with data available at all three ages (see Appendix A). All infants were Caucasian from English-speaking environments, mid to low-mid SES, and typically developing with no known risk factors.

All the infants were part of a longitudinal study of vocal development on typically developing infants. Parents of the infants were recruited through child-birth education classes and word of mouth for the longitudinal study. Interested individuals were given a consent form and questionnaire. Families returning the questionnaire and meeting inclusion criteria were contacted for an interview. All procedures were approved by The University of Memphis Institutional Review Board for the Protection of Human subjects.

### Recordings and recording procedure

The battery-powered, palm-sized LENA recorder was placed in the chest pocket of special infant clothing, with the microphone 7–12 cm from the infants' mouths. The recorder allowed us to investigate the naturalistic language environment conveniently with recordings up to 16 h/day at high sound quality, 16 kHz sampling rate (Xu et al., 2008). Parents were instructed by laboratory staff about how to place and activate the LENA recorder in the pocket of infant clothing at home. The parents brought the recorder to the laboratory after completing recordings according to a prescribed schedule, and laboratory staff uploaded the recordings through the LENA software. Once recordings were uploaded, automated analysis through the LENA software provided an estimated rate of infants' speech-like vocalizations (i.e., protophones) during each 5-min.

As a part of the longitudinal study, there were LENA all-day home recordings available for most of the 12 infants at each of the ages of 0, 1, and 3 months, that is during the first, second and fourth months of life—29 recordings in all (see Appendix A where the table indicates the 7 missing recordings). In a prior effort, 34 five-min segments from each infant had been selected for human coding for each of the 29 recordings (Oller et al., 2014; Yoo et al., 2014). In order to obtain representative segments across each day, 24 of the 34 segments had been selected at equal intervals across each recording day. The researchers had also chosen the 10 segments with highest volubility (infant vocalization count) for each recording based on the automated estimates of the LENA software. That is, we rank-ordered all the 5-min segments for the recording in terms of the counts of infant vocalizations estimated by LENA and selected the 10 segments with the highest counts.

All the selected segments (34 per infant per age) had been coded in real time by trained human coders. Given that there were 29 recordings, there were 986 coded segments available. Each infant utterance was categorized as a protophone [squeal, growl, vocant (i.e., vowel-like sound)], cry, or laugh. Coders also indicated in response to a questionnaire after coding each 5-min segment, how much of the time on a five-point scale, caregivers were talking to their infants.

To investigate caregiver *responses* to infant vocalizations in the present study, we selected 290 segments out of the 986 that had been previously coded: the selected segments were required to have (1) some infant-directed-speech (IDS), according to the questionnaire answered by coders at the end of each coding session, and (2) a high rate of protophone or cry as determined by the prior coding. We selected the 5 segments for each recording that had the highest protophone rates along with the 5 segments for each recording that had the highest cry rates (see Appendix A). This procedure constitutes a compromise between selecting completely random samples across the day (for maximal representativeness) and selecting for samples with sufficient numbers of infant vocalizations and parent responses to power our proposed analyses.

On the five-point scale of the questionnaire, “1” indicated that no one was talking to the infant during the 5-min and “5” indicated that someone was talking to the infant close to the whole 5-min. Segments that were marked “2” (less than half the time) or higher on the questionnaire were designated as candidates for selection. To avoid too many empty cells in the design, additional human listening was conducted to seek indications of IDS even in cases where the questionnaire responses had indicated 1 (no one talking to the infants). The original coding had been done in real time, and so the coders may have failed to notice some IDS. The new coding was conducted in repeat-listening (coders were allowed to listen to the same periods several times). Twelve percent of the 290 selected segments were included in the study based on this additional human listening, which determined that there were indeed some IDS utterances in those segments where the questionnaire data had not indicated that IDS was present. Still, 18 out of the 290 segments (6.2%) had no cases of IDS responses to infant utterances. Appendix B summarizes the available data. See below for definition of IDS responses.

### Coding and measurement

The coding team consisted of 4 Masters students and 1 PhD student in Communication Sciences and Disorders. In several intensive training sessions (with the last author, who has trained coders in infant vocal development for more than 40 years) of about an hour and a half each, all coders were introduced to how to locate boundaries for infant protophones, infant cries and caregiver utterances in AACT (Action Analysis, Coding, and Training, Delgado, 1996) software according to coding criteria listed below.

After training, the 5-min segments were coded by the five coders in repeat-listening mode to locate onset and offset of each vocalization. This coding procedure allowed us to measure lag times between each infant vocalization and any responsive caregiver vocalization. To locate utterances, we applied the breath-group criterion suggested by Lynch et al. (1995). According to the criterion, one utterance consists of a vocalization occurring on one egress (one expiration) and a new utterance can begin after each inspiration. We used the breath-group criterion because speech is organized in groups of expiration accompanied by phonation and supraglottal articulation, and because this criterion has proven to yield better intercoder agreement than methods based on fixed time intervals of silences (Lynch et al., 1995).

In order to quantify temporal structure of caregiver vocal responses, we first needed to identify cry as opposed to protophones. Protophones are defined as flexibly produced vocalizations including vowel-like sounds, squeals, growls, and so on (Oller, 2000). Cry conveys distress and always expresses negative affect whereas protophones are considered to be precursors to speech, not being bound to specific affect (Scheiner et al., 2002; Oller et al., 2013). Thus, cries are bound to a fixed affective state (i.e., negative) whereas protophones are not bound in this way. Protophones can be produced with different affect (i.e., positive, negative, and neutral) on different occasions. For example, infants can produce squeal (high pitch) sounds with positive affect in a joyful state and the same sounds with negative affect in a distressed state. This variability in usage of protophones (but not cries) is called functional flexibility (Oller et al., 2013). The distinction in functional flexibility between cry and protophones is important because we hypothesized that caregivers would respond differently to cry and protophones. We reasoned that cry is a signal for eliciting caregiver attention and aid, whereas protophones may be more likely to elicit pure social interaction.

Coders were trained to recognize markers for cry in terms of intense nuclei, dysphonation, glottal bursts and catch breaths (Truby and Lind, 1965; Stark et al., 1975). Appendix C provides a few example spectrographic displays and accompanying waveforms (Audios 1–3). Very intense cries are easy to identify and agree upon. They tend to have very intense, long dysphonated nuclei. They sometimes include glottal bursts or catch breaths at the beginning or end of each utterance. Utterances with glottal bursts or catch breaths are sometimes interpreted as negative even though they have less intense or short nuclei. Coders were trained to recognize one such common negative sound, which we term whimper, as displayed in Appendix C. After this training we found excellent agreement among coders as reported below.

Each caregiver utterance was identified as being infant-directed speech (IDS) or adult-directed speech (ADS). These identifications were quite reliable, because they were based on special phonatory characteristics of IDS, and because the meaning of both IDS and ADS was often clear to the listeners. In fact, the meaningful content usually made it totally unambiguous whether the parent was talking to the baby or not (e.g., “oh, you're the cutest little thing today” or “let's change your diaper now”). IDS has often been called “motherese” or “baby talk” because (in addition to special meaningful content) it often includes unique phonatory characteristics such as wide pitch range, high pitch, smooth intonation, and long duration per syllable. A recent study by Farran et al. (2016) reported that IDS utterances are identifiable with intercoder agreement > 0.9 as measured by Intraclass Correlation, and our data (see below) confirm very high agreement levels among coders. We identified each utterance of adults as IDS from parents, IDS from other adults, or ADS. For the purposes of the present study, however, only IDS from *parents* was used in determining timing relations with infant utterances.

### Calculating lag time

To address the hypotheses of the present study, we measured how fast and how often caregivers responded vocally to infant vocalizations. We follow a tradition (based on the floor transfer offset, for review see Holler et al., 2015) where lag is treated as the relation between the offset of one individual vocalization and the onset of another individual vocalization within a limited frame. In our approach, one infant utterance and one caregiver utterance are paired, the caregiver utterance being referred to as the response. Positive lag occurs when a caregiver vocal response begins after the paired infant vocalization offset (but within 5 s). Negative lag occurs when a response begins before the infant vocalization is over. Positive lag can be viewed as suggesting turn taking, because there is no overlap.

By our definition only one response can occur to an infant utterance, and that response must be the first caregiver utterance that meets the timing requirements. Also a caregiver utterance can be considered a response to one and only one infant utterance, namely the last infant utterance in time with respect to which the caregiver utterance meets the timing requirements. We included in the data analyzed below, the response lags for all the pairs of infant and caregiver utterances that met these requirements within the recordings.

**Figure 1:**
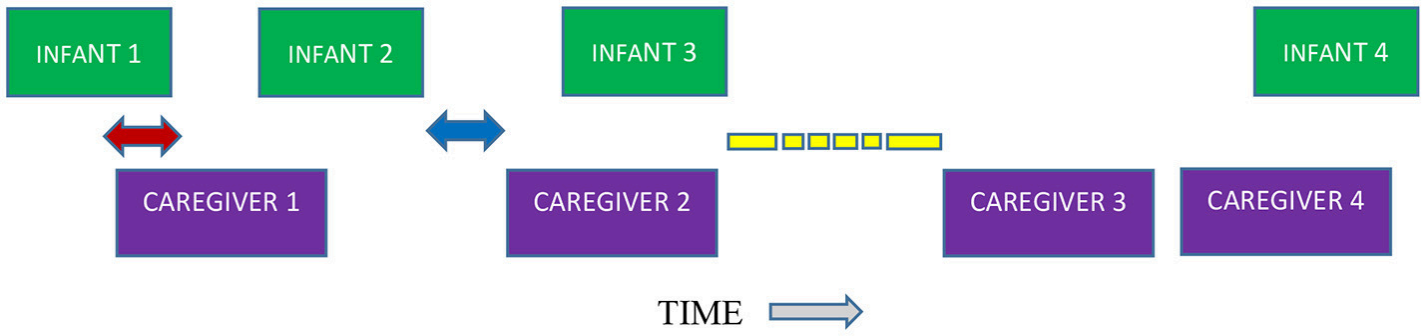
Calculating lags as the relation between offset of infant utterances/vocalizations to onset of caregiver utterances/vocalizations. Green blocks represent 4 infant vocalizations arranged in time. Purple blocks represent 4 caregiver vocalizations arranged in time. The red arrow indicates that infant vocalization 1 is overlapped with caregiver vocalization 1, showing negative lag. The blue arrow, on the other hand, shows alternating of caregiver vocalization 2 with infant vocalization 2, positive lag. The broken yellow bar represents a time period longer than 5 sec. If a caregiver vocalization occurs > 5 sec after the offset of an infant vocalization (as in the relation between infant vocalization 3 and caregiver vocalization 3), the caregiver vocalization is not defined as a response. Also, because caregiver vocalization 4 begins before the onset of infant vocalization 4, no vocal response to the infant vocalization is counted, even though the two vocalizations are overlapped. Similarly, caregiver vocalization 2 is a response to infant vocalization 2 but not to infant vocalization 3. In accord with our method, a caregiver vocalization can be assigned as a response to one and only one infant vocalization, and an infant vocalization can only be assigned to one responsive caregiver vocalization. Consider caregiver vocalization 2 with respect to infant vocalizations 1 and 2; if the duration from the offset of infant vocalization 1 to the onset of caregiver vocalization 2 is less than 5 sec, then a decision must be made about assignment. First, caregiver vocalization 2 cannot be assigned to infant vocalization 1 because caregiver vocalization 2's onset is closer in time to the onset of infant vocalization 2 than to the onset of infant vocalization 1 and thus must be assigned to infant vocalization 2. In addition infant vocalization 1 must be assigned to caregiver vocalization 1 and thus leaves no option for caregiver vocalization 2 to be assigned to infant vocalization 1.

Positive and negative lag values were measured in TF32, a flexible real-time acoustic analysis program with both waveform and spectrographic displays (Milenkovic, 2015). Cursors were placed at the beginning (onset) and end (offset) of each infant vocalization, and at the onset and offset of each caregiver IDS utterance, using the waveform displays supplemented (especially in cases of overlap) by narrow-band spectrographic displays that facilitated discrimination between the caregiver and infant voices. For the purposes of the present study, we only included the first caregiver responses within 5 s of infant vocalization offset. In Figure 1, we illustrate the principles for determining lags of caregiver vocal responses. We emphasize that each event represented by a green or purple box is an utterance (vocalization), defined by the breath-group criterion (see above). We measured timing of each single caregiver response to each single infant vocalization (either protophone or cry), *not* to a sequence of infant vocalizations. That is, we treated each infant vocalization and each caregiver vocalization separately.

### Coding and measurement agreement

For coder agreement tests, 28 out of the 290 segments were randomly selected: 6 segments at 0 months, 15 segments at 1 month, and 7 segments at 3 months. Each of the 5 coders coded all the 28 segments in repeat listening mode (just as coders did during primary data collection), locating the onset and offset of each utterance of infants and caregivers. Intraclass Correlation Coefficients (ICC) were calculated to assess inter-rater agreement on cry, protophone, and IDS. The average ICC for cries was 0.92 with a 95% confidence interval from 0.85 to 0.96 [*F*_(27, 108)_ = 85.2, *p* < 0.001]. In the case of protophones, the average ICC was 0.87 with a 95% confidence interval from 0.74 to 0.94 [*F*_(27, 108)_ = 61.2, *p* < 0.001]. A high degree of inter-rater agreement was also found in identifying IDS. The average ICC was 0.93 with a 95% confidence interval from 0.88 to 0.96 [*F*_(27, 108)_ = 71.2, *p* < 0.001]. Pearson correlations for each vocal type between all the possible pairings of coders were also calculated (M = 0.94, range: 0.89 to 0.98).

The temporal relation between infant and caregiver utterances is the primary research question of the present study, and so we determined the extent to which the coders identified similar patterns of relative timing between infant and caregiver utterances. With the 28 segments, we calculated mean response lags of caregiver utterances to infant cries as well as those to infant protophones (see section Results).

### Statistical analysis

Generalized Estimating Equations (GEE) were implemented in R to model lag time as a function of various covariates. GEE models are an extension of Generalized Linear Models (GLM) (McCullagh and Nelder, 1989). GLM are useful to account for dependent variables (DVs) that do not meet the assumptions that DVs are normally distributed and linearly related to predictors. GEE were proposed by Liang and Zeger (1986) to account for correlated, in other words, nested or clustered DVs. GEE models are also flexible for handling missing data as well as a variety of outcome variable distributions (Zeger et al., 1988).

As explained earlier, 5-min segments were selected based on rate of occurrence of infant protophones and cries that had been determined in the original coding from the prior study. This provision resulted in nesting (or clustering) of the data within each infant. In addition, 6.2% of the segments had no IDS, and thus constituted missing data. Also 5 of the 12 infants had no recording for at least one age and thus the data were not equally balanced across the infants (see Appendix B).

Independent and dependent variables used in GEE models for the study are summarized in Table 1. Various combinations of covariates, including interaction terms, were tested to find a good model fit for the data and the variables in the final model, which had the following form: Lag = Age + Vocal Type + Duration (Infant vocalizations). This model was chosen because it was associated with the only significant effects. We initially tested Birth order on the assumption that first-born infants may receive more caregiver responses (Downey, 1995), but this variable was dropped in the final model. Similarly we tested for Caregiver vocalization duration, because it seemed possible that infant vocalizations might be influenced by the duration of caregiver vocalizations. But again, this factor showed no notable effects on the dependent variable and was dropped in the final model.

**Table 1:** Variables used in the GEE model.

**Variables**		**Description**
Independent variables	Age	Infant age in months
	Vocal Type	Vocal type of infant utterance: protophone or cry
	Duration (Infant Vocalizations)	Utterance duration of protophone or cry
	Duration (Caregiver Vocalizations)	Utterance duration of IDS
	Birth order	Birth order of each infant
Dependent variable	Lag	Time difference between offset of infant utterance and onset of caregiver utterance

## Results

### Infant and caregiver vocalizations in naturalistic environments

The average percentage of infant utterances that were responded to with IDS in these segments selected from all-day recordings ranged for the three ages from 10 to 21% for protophones and from 13 to 17% for cries (Table 2, and for more details see Appendix B). In contrast, in laboratory studies with infants as young as 3 months, the percentage of infant utterances with responses has been much higher (generally more than 50% responses), presumably because in the laboratory, caregivers have usually been *instructed* to interact with infants and have stayed always in the same room with the infants (review in Fagan and Doveikis, 2017).

**Table 2:** Infant and caregiver vocalizations in the segments selected from the all-day recordings.

**Infant**	**Sum of IDS and ADS utterances**	**No. of IDS**	**No. of IDS responses**		**Mean proportions of IDS responses to infant vocalizations**	
			**To protophones**	**To cries**	**To protophones**	**To cries**
0 months	2191	1697	778	355	0.15	0.17
1 month	1495	1493	626	259	0.10	0.13
3 months	2234	2234	1129	111	0.21	0.15

*IDS, Infant-Directed Speech; ADS, Adult-Directed Speech*.

To see how much IDS was produced within the 5-min segments from all-day recordings, we summed durations of all IDS within each segment. Then, mean, median, min, and max of IDS durations at each age were calculated (Table 3). On average about 10% of the time within the 5-min segments was occupied by IDS. In contrast prior results based on short-term recordings where caregivers have been instructed to interact with infants have shown from 40 to 70% of the time occupied by IDS (e.g., Gros-Louis et al., 2006; Kärtner et al., 2010).

**Table 3:** Duration and percent of caregiver IDS within 5-min segments across infants at each age.

	**IDS Duration (s) during 5 min**				**Percent (%) of 5 min**			
	**Mean**	**Median**	**Min**	**Max**	**Mean**	**Median**	**Min**	**Max**
0 months	29.3	14.8	0	140.1	9.8	5	0	47
1 month	23.3	9.2	0	150.6	7.8	3	0	50
3 months	38.6	21.6	0	161.7	13	7.2	0	54

In sum, caregiver responsivity was very different in our naturalistic environments compared to prior results obtained in structured laboratory environments. In our data caregivers tended to produce less IDS and consequently responded less to infant vocalizations than in studies where parents were instructed to interact with their infants in a laboratory (or even at home).

### Temporal structure of caregiver IDS in response to protophones and cries

**Figure 2:**
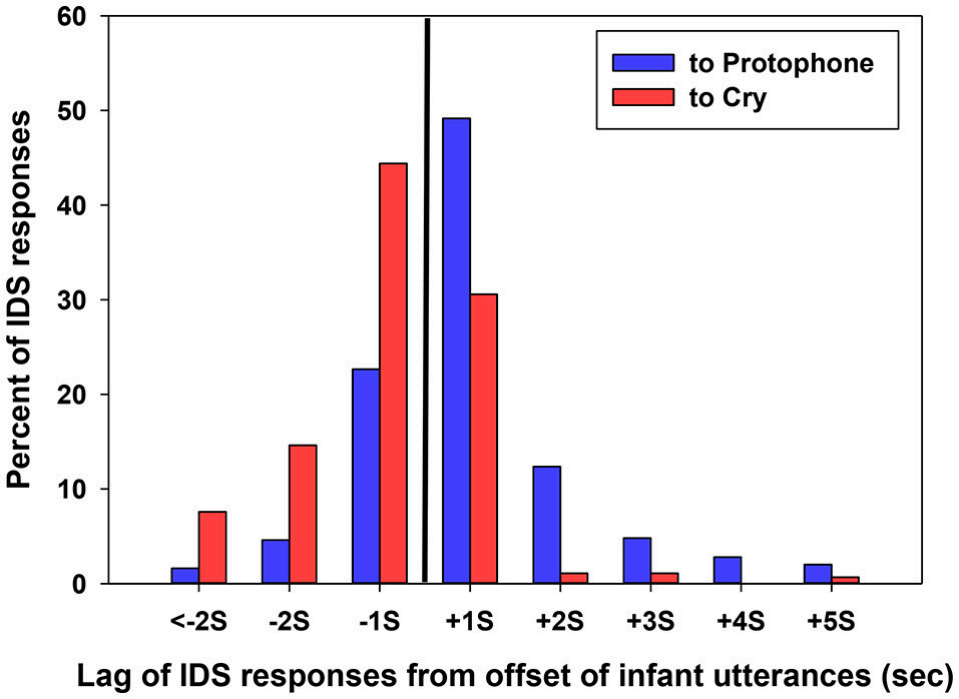
The black vertical line represents the offset of infant utterances, the 0 point in time. Percent of all IDS responses to cries and protophones is plotted for each 1-s interval before and after the 0 point. The display shows that IDS utterances in response to protophones tended to begin *after* the offset of infant utterances (positive lag), and especially in the 1 s interval after. In contrast, IDS in response to cry tended to begin *before* the offset of infant utterances (negative lag), overlapping with them.

Figure 2 shows proportions of IDS utterances in response either to protophones or cries in 1 second intervals referenced with regard to the offset of infant utterances after collapsing the data across ages. The vertical line in the Figure indicates the point of offset of infant utterances, “0” on the x-axis. Thus, percent of IDS utterances beginning in each interval after the offset is displayed right of the black line, and each interval in seconds is labeled “+” on the x-axis, indicating positive lag. Similarly, percent of IDS utterances beginning in each interval before the offset is displayed left of the black line, and values in seconds are labeled with a minus sign on the x-axis, indicating negative lags. The figure displays a range from < −2 s to > +5 s lag. Long negative lags were rare, as indicated in the figure, because infant utterances were usually not long enough to allow them. For data collapsed across all three ages, 71% of IDS responses to protophones began *after* the offset of infant utterances whereas 66% of IDS responses to cries began *before* the offset of infant utterances.

Distributions of the data in Figure 2 also show that IDS either to protophones or cries was heavily concentrated within the 1 second interval around the offset of infant utterances and became sparse as lags increased positively or negatively. Short latency of caregiver responsivity has been suggested by Papoušek and Papoušek (1987) and Keller et al. (1999), although neither prior study nor any other prior one to our knowledge has distinguished between lags of responses to protophones and cry. The present study confirms previous findings overall, but adds the clarification that a preponderance of responses occurring in the first second *after* offset of infant vocalizations applies to protophones, but *not* to cries. This pattern of results applied to all the coders in the agreement data. For the 28 segments that were coded by all of them, the mean lag for each of the coders was positive and occurred within the first second after the infant offset (in fact the first half second) for protophones, and mean lag was negative and occurred within the first second before the infant offset for cries (Table 4).

**Table 4:** Coder agreement on response lags to infant vocalizations.

	**Mean response lags to**	**Mean response lags to**
	**protophones (ms)**	**cries (ms)**
Coder 1	463	−133.94
Coder 2	446.25	−229.62
Coder 3	423.82	−334.7
Coder 4	362.75	−173.54
Coder 5	417.25	−229.53

**Figure 3:**
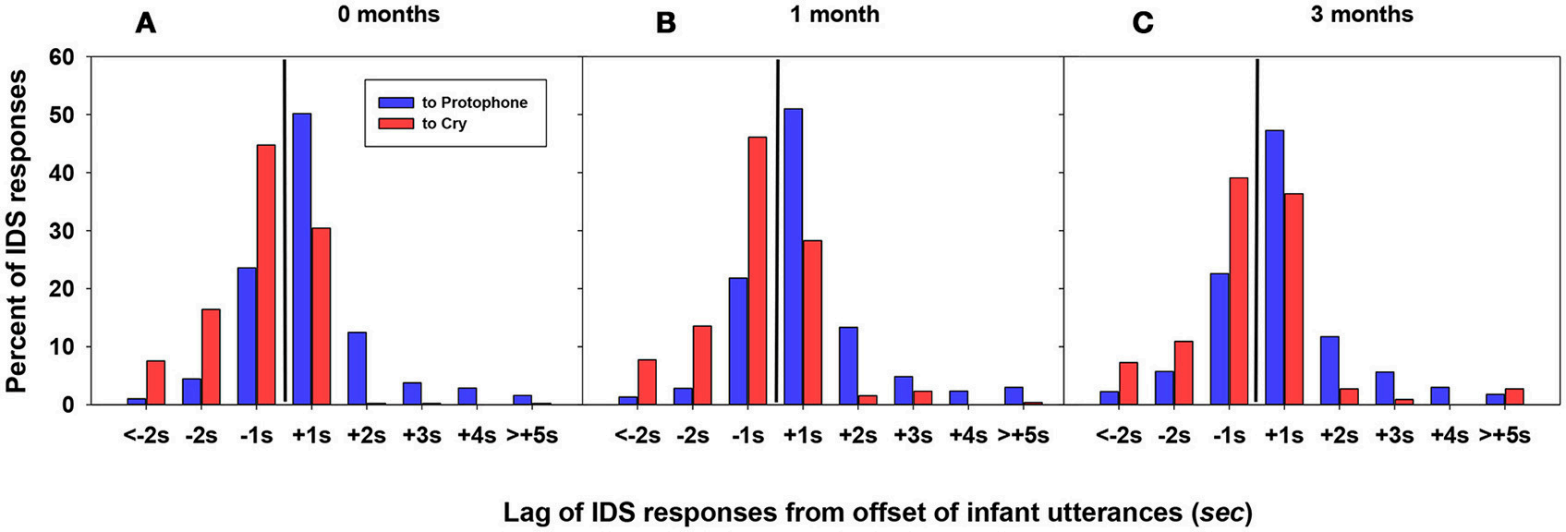
**(A)** Lag of IDS responses for infants at 0 months. **(B)** Lag of IDS responses for 1 month. **(C)** Lag of IDS responses for 3 months. For all three panels, as in Figure 2, the black vertical line represents the offset of infant utterances. The display shows that IDS in response to protophones tends to being after the offset of infant utterances (positive lag), and especially in the 1 s interval after. In contrast, IDS in response to cry tends to begin before the offset of infant utterances (negative lag), overlapping with them. This pattern is consistent at each age.

Breaking the data down by age, as shown in Figure 3, a similar distribution of lags to protophones and cries was observed at each of the three ages, with higher proportion of responses near the offset of infant utterances at all ages. Caregivers responded to protophones mostly after the offset of infant utterances whereas they responded to cries mostly before the offset of infant utterances. At 0 months, IDS responses to *protophones* occurred in 71% of the cases after the offset of infant utterances, whereas IDS to cries occurred 69% before the offset of infant utterances. At 1 month, IDS to protophones occurred 74% after the offset of infant utterances, whereas IDS to cries occurred 67% before. At 3 months, IDS to protophones occurred 69% after the offset of infant utterances whereas IDS to cries (which occurred very infrequently at 3 months) occurred 57% before the offset of infant utterances.

**Figure 4:**
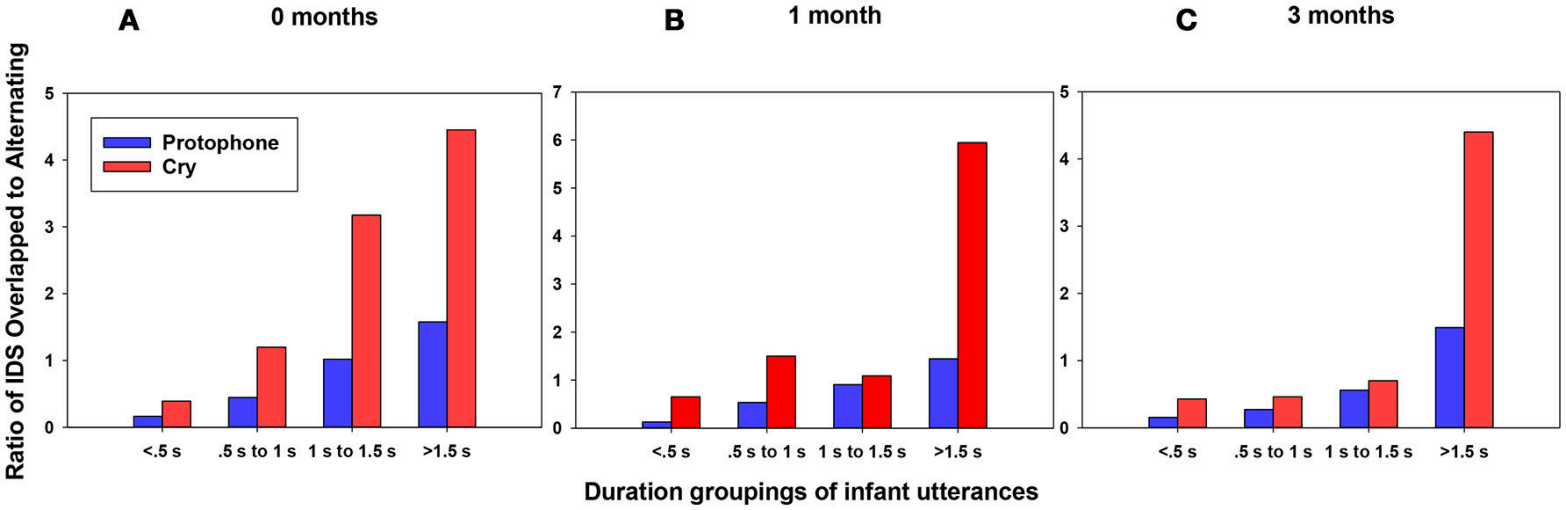
**(A)** Duration groupings for infants at 0 months. **(B)** Duration groupings for 1 month. **(C)** Duration groupings for 3 months. For all three panels, degree of overlap/alternation of caregiver responses to protophones and cries in groupings of 0.5 s (i.e., 500 ms bins). The display shows that regardless of duration of infant utterances, either cry or protophones, caregivers tended to respond to infant cry with more overlap (higher ratio of overlapped/alternating) than to protophones. Conversely, turn-taking (lower ratio of overlapped/alternating) tended to occur to a greater extent with protophones than with cries at all durations of utterances. The display also shows that ratios of overlap to alternation were higher at longer durations of infant utterances for both cries and protophones, with a very high ratio for long cries.

A possible artifact in the data needs to be considered. Namely, cries in the data were more than twice as long on average as protophones^2^. Could it be that the tendency for IDS to overlap with cries more than with protophones was an artifact of this difference in mean durations? To test for this possibility we segregated the data for both cries and protophones into 500 ms bins^3^, and plotted proportion of overlapped to alternating IDS (the ratio of overlapped caregiver responses to alternating caregiver responses) as shown in Figure 4. Regardless of duration of infant utterances, caregiver responses overlapped more often with cries than with protophones. The pattern applied at all ages and at all durations (Figure 4). The statistical significance of the tendency for alternation to protophones as opposed to overlap with cries was tested by Chi-Square, with significant findings in 9 of the 12 comparisons (Table 5). The analyses suggest that the duration differences between cries and protophones were not responsible for the differentiation in IDS lags for cries and protophones. On the other hand, duration was not irrelevant in the pattern of IDS responsivity. The maximum difference in the ratios in Figure 4 was observed for the longest utterances (>1.5 s), both for cries and protophones, and in general there was a tendency for more overlap of IDS at longer durations. Thus, the data suggest that the longer the infant utterance (whether protophone or cry), the less likely caregivers were to produce their IDS response after the infant utterance was finished.

**Table 5:** Chi-Square statistics for alternation vs. overlap for protophones and cries at various durations of infant utterances.

	**< 500 ms**	**500 ms to 1 s**	**1 to 1.5 s**	**>1.5 s**
0 months	4.52^*^	16.44^**^	11.71^**^	14.71^**^
1 month	23.97^**^	8.84^**^	0.23	16.85^**^
3 months	4.35^*^	1.06	0.19	9.03^**^

* *p < 0.05*,

** *p < 0.01*.

A GEE model confirmed the predicted patterns of positive lag for IDS in response to protophones vs. negative lag for IDS in response to cries, taking account in the model for the clustered data. Among variables summarized in Table 1, age (0, 1, or 3 months), vocal types (cry vs. protophone), and duration of infant vocalizations (treated as a continuous variable) showed significant main effects, while birth order, age-vocal type interaction, and duration of caregiver IDS utterances were not significant in the model (Table 6). The GEE model predicted that as infant age increased, lag of IDS increased. With regard to vocal types, lags were positive when protophones were responded to with IDS but negative when cries were responded to. Duration of infant vocalizations showed a significant main effect in the GEE model. However, as shown in Table 6, since the coefficient of duration of infant vocalizations was extremely small, the duration of infant vocalizations was not, *in practical terms*, significantly associated with lag in the model.

**Table 6:** Significant parameters from the GEE analysis.

	**Coefficient**	**S.E**	***p*-value**
Intercept	−1.12	329.14	0.997
Age	14.39	6.72	0.032
Vocal Type (Protophones vs. Cries)	363.59	38.41	< 0.0001
Duration of Infant Vocalizations	−0.64	0.03	< 0.0001

Birth order did not show any significant main effect in the model. Birth order was included because prior research has suggested lower parent interaction with later borns (e.g., Downey, 1995) and because we observed that some recordings with low IDS were conducted with infants who had older sibling(s). Caregiver utterance duration was also not significant in the model. In other word, timing of caregiver responsivity to infant vocalizations was independent of caregiver utterance durations.

These findings provided evidence that caregiver IDS in response to protophones showed a turn-taking pattern even at 0 months. However, caregivers responded much differently to cries, overlapping rather than taking turns. Importantly, the distinctively different interaction patterns from caregivers to cries and protophones were observed even at 0 months, and the patterns remained similar at all three ages.

## Discussion

The development of vocal language appears to depend on both a capacity and an inclination of infants to vocalize plentifully and for caregivers to take advantage of those infant sounds to engage them in vocal interaction (Bruner, 1983; Bornstein and Bruner, 2014). Many have noticed the tendency of caregivers to interact with their infants vocally (Bell and Ainsworth, 1972; Richman et al., 1992; Keller et al., 1999), but a key opportunity to illuminate the process has not previously been exploited. The opportunity resides in the difference between cry sounds of the human infant and the precursors to speech, the protophones. We hypothesized, in agreement with Stern et al. (1975), that cry sounds should not elicit alternating caregiver vocal responses, because cry sounds are not the potential material of speech. To the extent that caregivers, even interacting with infants in the first month of life, intuitively alternate their vocalizations with protophones, but overlap their vocalizations with cries, they provide compelling evidence that human caregivers are predisposed to treat protophones as potential speech material long before infants are capable of speaking. Our results empirically confirm Stern's suspicion and our own, as caregivers were far more inclined to converse in alternating fashion with protophones than with cries^4^.

The results, we think, offer an enhancement to prior perspectives on the importance of early vocal interaction, because they illustrate that human caregivers must possess not only a capacity to recognize protophones as primitive speech material, but a predisposition to treat the protophones as such by interacting with them in a protoconversational way. The contrast in the way caregivers in our research reacted to protophones and cries highlights the fact that caregivers know, even if subliminally, that protophones offer a special opportunity to bond with the infant and to set the process of speech development on course.

We found that caregiver vocal responses to protophones were heavily concentrated in the 1 sec interval after the offset of infant protophones. This finding is consistent with the results of Keller et al. (1999), studying interactions with infants at 3 months, showing that maternal responses (verbal or non-verbal) occurred most frequently within the 1 s after infant behaviors occurred. Papoušek and Papoušek (1987) suggested caregivers' contingent responses to infant vocalization occurred within 800 ms. Infants seem to be capable of perceiving contingency from birth (Murray and Trevarthen, 1985; Gewirtz and Pelaez-Nogueras, 1992; Striano and Reid, 2006). According to Keller et al. (1999), “the experience of contingency allows the infant to develop expectations about behavioral occurrences …” (p. 475). Caregiver responses to the protophones thus appear to provide a rich learning opportunity. Of course coaction with parent and infant vocalization in unison does appear to occur on occasion even with the protophones. The pattern of coaction may reflect another function of interactivity that, although it occurs infrequently, may be of considerable importance in child development.

While many longitudinal studies have shown that protophones are foundations for speech (Oller, 1980; Stark, 1980; Koopmans-van Beinum and van der Stelt, 1986; Roug et al., 1989), some still assert that protophones develop *from* cries (Mampe et al., 2009; Takahashi et al., 2015), and thus imply that protophones are absent in the first months of life. In fact, however, infants produce *both* protophones and cries from birth (e.g., Nathani et al., 2006). Moreover, the evidence shows that, protophones occur *more frequently* than cries, even in the first 2 months, and that the preponderance of protophones over cries increases to a ratio of perhaps 8 to 1 by 3 months and continues to expand thereafter. This evidence in itself suggests that failure to recognize the significance of protophones from birth may have misled prior theorists. The modern evidence suggests a massive endogenous tendency on the part of infants, from the beginning of life (Oller, 2000; Nathani et al., 2006; Jhang and Oller, 2017), to explore the vocal capacity with protophones. Infant vocal exploration thus offers caregivers a basis for laying a frame for bonding and social interaction with infants and for protoconversation as an expression of the caregiver investment in the relationship with infants. Significant consequences for potential language learning seem obvious even if neither the caregiver nor the infant has any immediate awareness of the long-term significance of their interactions.

There exists persuasive empirical evidence that caregivers' intuitive interaction with these infant vocalizations is highly associated with cognitive and language development (Lewis and Goldberg, 1969; Ainsworth and Bell, 1974; Lewis and Coates, 1980; Jaffe et al., 2001). Surprisingly, however, cries and/or distress sounds have been almost entirely ignored in prior face-to-face interaction literature that has attempted to address the role of interaction in language development—responses to cries and fussing sounds have typically not been coded at all in such studies. Stern et al. (1975) had speculated that caregivers usually speak to infants simultaneously with their cries, and consequently had brought into focus the opportunity to illustrate the power of the protophones to elicit conversational reactions. But Stern's speculation requires that reactions to protophones be systematically contrasted with reactions to cries. Given his extensive influence on the literature, we are surprised that no empirical demonstration of this distinction in caregiver reactions has been made until the present work.

While the many prior results suggest that early protophones are foundations for speech, it is notable that their form is very distant from the form of speech, particularly because early protophones do not consist of well-formed (“canonical”) syllables. Canonical syllables, not produced systematically until the second half year, have long been recognized as speech precursors, because there exists a clear continuity between canonical syllables and early meaningful speech—the types of syllables utilized in the two cases are very similar (Oller et al., 1976; Vihman et al., 1985; Locke, 1989; Stoel-Gammon, 1989). And when the canonical stage begins, caregivers react not only by interacting with infants in protoconversation, but saliently by treating the canonical syllables as potential words (e.g., Papoušek, 1994). A canonical babble sequence [dada] can immediately be treated as “daddy,” even though the infant presumably didn't intend it that way. In contrast, the early protophones are rarely if ever treated by caregivers as possible words.

As early as the 1970's the precanonical protophones were already recognized as being related to speech because of their tendency to include normal phonation (the kind of phonation that is overwhelmingly predominant in speech) and because the primitive articulation patterns that often accompany early protophones hint at a foundation for speech articulation (Zlatin, 1975; Oller, 1981; Stark, 1981). More recently, precanonical protophones have also been recognized as foundations for speech because they (unlike cries) possess functional flexibility, which is a fundamental property for all natural languages (Oller, 1981; Scheiner et al., 2002; Oller et al., 2013; Iyer and Ertmer, 2014). The present results indicate that caregivers intuitively provide systematic conversational frames in response to precanonical protophones, even at 0 to 3 months, thus introducing the infant to the turn-taking system that characterizes most speech interaction.

In our data, caregivers responded to cries at about the same rate as to protophones (see Table 2), but there were many more protophones available for response, so the data consisted primarily of responses to protophones. A question that arises is why caregivers respond vocally to cries at all, since they are not natural speech material. Stern et al. (1975) contended that “.mothers commonly vocalize simultaneously with the crying of their infants in order to soothe them” (p. 90). The idea finds partial support in the suggestion of Wolff (1965) that continuous sound (particularly white noise) can soothe neonates. Bell and Ainsworth (1972) reported that caregiver vocal responses (without touching the baby) to cries were the second most common responses to cries, following physical responses (pick-up and hold the baby). Interestingly, however, mere vocal responses to cries were found to be the least effective intervention to terminate cries. In the face of these results and interpretations it is not clear whether caregivers in prior work or in our own were using simultaneous speech over cries principally to soothe infants. This is a question that could be investigated productively with audio-video recorded interactions.

Another focus of our investigation is caregiver responsivity in a much more naturalistic environment than in most prior research on interaction. We found that caregivers tended to respond much less often to infant vocalizations in all-day recordings compared to prior research conducted in structured settings. On average caregivers responded in our study to 10–21% of infant vocalizations in 5-min segments. In contrast, Kärtner et al. (2010) reported that on average, mothers contingently responded to infant non-distress vocalizations at a rate of 47% in 10 min structured interactions. Gros-Louis et al. (2006) reported even higher maternal contingent response rates to infant vocalizations: 73% in 10 min play sessions. Fagan and Doveikis (2017) reported that mothers responded to about 30% of infant utterances in ordinary interaction at home, while they summarized prior literature suggesting laboratory rates in structured interactions of about 70%. Although Fagan and Doveikis did not obtain their data with all-day recordings, their motivation and results are consistent with ours. When mothers are instructed to interact, their voices often occupy a considerable portion of the total time of observation. Franklin et al. (2014) found that in face-to-face interaction with 6-month olds in the “still-face” paradigm, mothers' speech occupied about 50% of the time. Dominguez et al. (2016) reported that mothers' speech occupied about 29% of the time in observations where their newborn infants were present and awake with them for 10 min. Farran et al. (2016) reported that mothers' speech occupied about 25% (during 10 min selected from home and laboratory recordings where mothers were expected to interact with their infants) in both Lebanese and American mother-infant dyads. In contrast Table 2 indicates that in our all-day home recordings only 8 to 13% of the time was occupied by caregiver responses to infant vocalizations.

Overall, the results suggest much lower rates of caregiver responsivity to infant vocalizations in our study than in laboratory studies, presumably because our interactions occurred in households where no experimenters instructed mothers to interact nor observed them doing it and where mothers were free to move about in various rooms in the house. We presume our results reflect more representative patterns of interaction, where caregivers in their natural environments choose convenient moments to interact with their infants, focusing on the special circumstance of interaction with protophones, fostering sociality, bonding, and laying groundwork for language.

## Author contributions

HY conceived the study, designed the study, coordinated the coders, analyzed the data, and wrote the paper. DAB conducted the statistical analysis and reviewed the paper. DKO designed the study, analyzed the data and wrote the paper.

### Conflict of interest statement

The authors declare that the research was conducted in the absence of any commercial or financial relationships that could be construed as a potential conflict of interest.

## Footnotes

^1^Cries can be subcategorized into high distress wail cries and lower distress whimpers (sometimes called “fuss” in the literature). In the present work, we did not in the original coding differentiate these cry types, but coded both types as cries.

^2^Cries in the data were more than twice as long on average as protophones (0 month: Prot = 742 ms, Cry = 1709 ms; 1 month: Prot = 660 ms, Cry = 1664 ms; 3 months: Prot = 1034 ms, Cry = 1697 ms).

^3^We collapsed infant utterances into 500 ms utterance duration groupings or “bins” for the analysis in Figure 4. That is, all infant utterances less than 500 ms were collapsed into one bin, all utterances between 500 ms and 1 s were grouped together in another bin, and so on. After collapsing them into these 500 ms bins, timing of caregiver responses was determined for each bin at each age and displayed as the ratio of the number of caregiver responses overlapping with infant utterances over the number of caregiver responses alternating with (following) infant utterances. By doing this, we tested whether duration of infant utterances affected the tendency for caregivers to overlap vocalization with cries and to alternate with protophones.

^4^In the present work, we did not, in the original coding, differentiate cry types, but coded both wail cries and whimpers as cries. However, after all the data had been preliminarily analyzed, we conducted an additional round of coding just in order to differentiate wail cries and whimpers in our samples. Whimpers turned out to be a relatively small percentage of all cries at 0 and 1 months of age (17–18%) but represented ~48% of cries at 3 months. Note also in Appendix B that the occurrence rate of both wail cries and whimpers was dramatically lower at 3 months than at the other ages. The N of whimpers that were responded to by the parents was very small, < 40 at each age, so the power of any analysis of them is very low. Nonetheless we computed mean lags of responses to wail cries and to whimpers and found that, collapsing data across the three ages, caregiver responses showed the expected pattern of more overlap to whimpers than to protophones, but the trend was not as strong as for wail cries. Further, the expected pattern occurred at all 3 ages for wail cries, but for whimpers it occurred only at 0 and 1 months.
