# Supplementary material for: The Origin of Protoconversation: An Examination of Caregiver Responses to Cry and Speech-Like Vocalizations
Source: Front Psychol. 2018 Aug 24;9:1510. doi: 10.3389/fpsyg.2018.01510 (PMC6117422; doi:10.3389/fpsyg.2018.01510)
Supplement: Supplementary file 1 [file Data_Sheet_1.pdf]

Appendix A

Infant	Gender	Birth Order	Age in months	# of 5 min segments selected for coding	
				Protophone	Cry
1	M	2	0	-	-
			1	-	-
			3	5	5
2	F	1	0	-	-
			1	-	-
			3	5	5
3	M	2	0	5	5
			1	5	5
			3	-	-
4	F	2	0	5	5
			1	5	5
			3	-	-
5	F	2	0	-	-
			1	5	5
			3	5	5
6	M	1	0	5	5
			1	5	5
			3	5	5
7	M	2	0	5	5
			1	5	5
			3	5	5
8	M	1	0	5	5
			1	5	5
			3	5	5
9	M	3	0	5	5
			1	5	5
			3	5	5
10	F	1	0	5	5
			1	5	5
			3	5	5
11	F	2	0	5	5
			1	5	5
			3	5	5
12	F	1	0	5	5
			1	5	5
			3	5	5

Appendix B

Infant	Age in months	# of infant utterances		# of caregiver utterances (IDS and ADS)	# of IDS utterances	# of IDS responses[a]		# of IDS responses/ # of infant utterances	
		Prot	Cry			To Prot	To Cry	Prot	Cry
1	0	-	-	-	-	-	-	-	-
	1	-	-	-	-	-	-	-	-
	3	719	90	383	383	237	41	.33	.46
2	0	-	-	-	-	-	-	-	-
	1	-	-	-	-	-	-	-	-
	3	356	12	337	337	111	5	.31	.41
3	0	631	29	45	44	26	2	.04	.06
	1	833	247	51	51	33	15	.04	.06
	3	-	-	-	-	-	-	-	-
4	0	659	401	305	200	107	82	.16	.20
	1	912	91	91	91	65	5	.07	.05
	3	-	-	-	-	-	-	-	-
5	0	-	-	-	-	-	-	-	-
	1	289	167	228	228	81	25	.28	.15
	3	387	204	138	138	46	19	.12	.09
6	0	730	569	302	291	90	70	.12	.12
	1	1149	684	209	207	107	60	.09	.09
	3	1013	26	392	392	243	2	.24	.08
7	0	477	90	43	22	15	1	.03	.01
	1	456	36	29	29	18	2	.04	.06
	3	288	21	112	112	37	5	.13	.24
8	0	808	287	399	399	228	66	.28	.23
	1	647	278	333	333	160	81	.25	.30
	3	868	73	393	393	275	18	.32	.25
9	0	849	31	167	6	6	0	.01	0
	1	474	1	148	148	20	0	.04	0
	3	635	58	31	31	21	1	.03	.02
10	0	142	30	266	266	77	16	.54	.53
	1	136	36	188	188	31	22	.23	.61
	3	295	100	157	157	47	2	.16	.02
11	0	477	297	261	85	63	16	.13	.05
	1	403	76	50	50	28	8	.07	.11
	3	525	50	100	100	33	12	.06	.24
12	0	537	371	403	384	166	102	.31	.27
	1	687	308	168	168	83	41	.12	.13
	3	412	115	191	191	79	6	.19	.05

IDS = Infant-Directed Speech, ADS = Adult-Directed Speech, Prot = Protophones

# of IDS utterances = total number of utterances of caregivers that were directed toward infants, some of which could be counted as responses to infant protophones or cries, and some of which were continuations of talk by the caregiver.

# of IDS responses = Number of caregiver IDS utterances that occurred as responses to infant protophones or cries; only the *first* IDS utterance in each caregiver sequence following the onset of an infant utterance was counted as a response. In addition any IDS utterance starting more than 5 sec after the offset of an infant utterance was not treated as a response.

[a] In 18 segments there were NO IDS responses even though there were infant protophones and/or cries and cases of IDS.

A minus sign (-) indicates that no recording was available for the infant at the designated age.

Appendix C

(A) Wail cry (high distress) with a strongly dysphonated portion of its nucleus

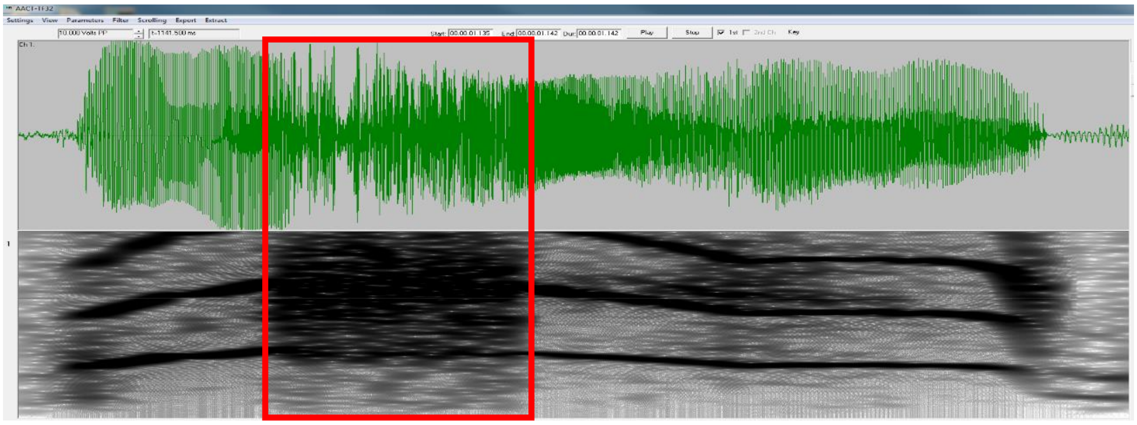

Dysphonated portion

(B) Wail cry with catch breath at the end of a nucleus including both normally phonated and dysphonated portions

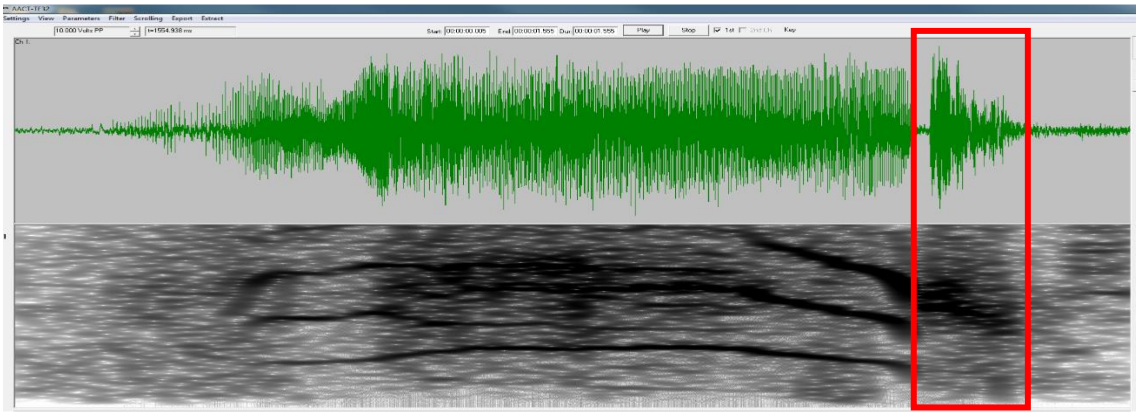

Rapid ingressive catch breath

(C) Whimper (low distress): nucleus and glottal burst

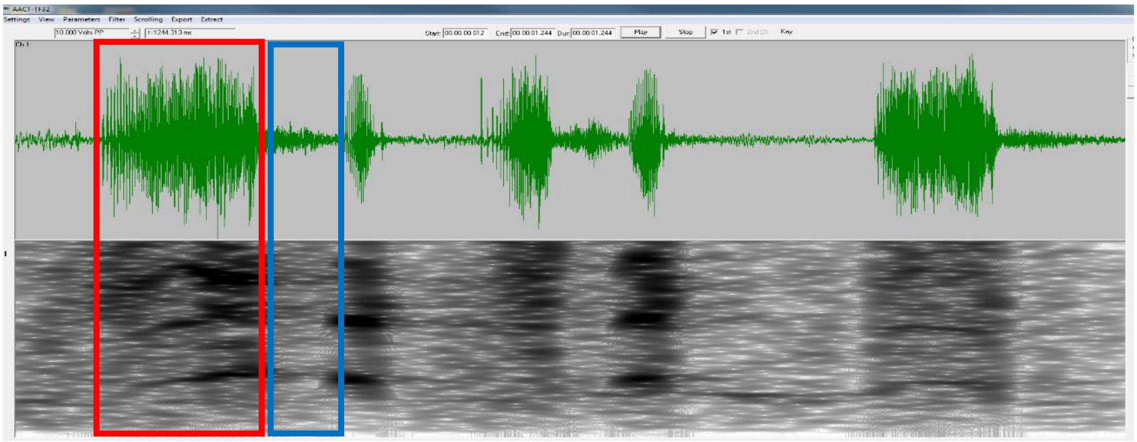

Nucleus

Glottal burst
